# Influence of Nanobubble Size Distribution on Ultrasound-Mediated Plasmid DNA and Messenger RNA Gene Delivery

**DOI:** 10.3389/fphar.2022.855495

**Published:** 2022-06-01

**Authors:** Hiroshi Kida, Loreto B. Feril, Yutaka Irie, Hitomi Endo, Keiji Itaka, Katsuro Tachibana

**Affiliations:** ^1^ Department of Anatomy, Faculty of Medicine, Fukuoka University, Fukuoka, Japan; ^2^ Department of Biofunction Research, Institute of Biomaterials and Bioengineering, Tokyo Medical and Dental University (TMDU), Tokyo, Japan

**Keywords:** gene transfection, sonoporation, ultrasound, nanobubble, gene therapy

## Abstract

The use of nanobubbles (NBs) for ultrasound-mediated gene therapy has recently attracted much attention. However, few studies have evaluated the effect of different NB size distribution to the efficiency of gene delivery into cells. In this study, various size of albumin stabilized sub-micron bubbles were examined in an *in vitro* ultrasound (1 MHz) irradiation setup in the aim to compare and optimize gene transfer efficiency. Results with pDNA showed that gene transfer efficiency in the presence of NB size of 254.7 ± 3.8 nm was 2.5 fold greater than those with 187.3 ± 4.8 nm. Similarly, carrier-free mRNA transfer efficiency increased in the same conditions. It is suggested that NB size greater than 200 nm contributed more to the delivery of genes into the cytoplasm with ultrasound. Although further experiments are needed to understand the underlying mechanism for this phenomenon, the present results offer valuable information in optimizing of NB for future ultrasound-mediate gene therapy.

## Introduction

Gene therapy has been intensively investigated as a forefront treatment for various cancers as well as for rare diseases. Therapeutic genetic materials such as DNA or RNA are required to reach the target cells in sufficient quantities to yield beneficial outcome. Recently, intramuscular injection of messenger RNA genes has demonstrated significant effectivity as a vaccine against coronavirus disease (Machado et al., 2021). It has increasingly become evident in the clinical situation that facilitation of therapeutic nucleic acid into cells is an important modality for the treatment of many diseases. Although use of viral vectors has proven to be the most efficient in clinical trials, non-viral type vectors are considered more promising from a safety standpoint. Consequently, novel non-viral gene delivery systems have been developed to avoid possible risk of immunogenicity, oncogenicity and inflammation potentiated by conventional viral gene vectors (Sainz-Ramos et al., 2021). These novel delivery systems include polymers and liposomes which carry genetic materials to the target site.

In the past 2 decades, a considerable amount of literature has been published on ultrasound mediated gene delivery. Sonoporation, which uses ultrasound to transiently increase cell membrane permeability, is a modality that has great potential to safely deliver genes into specific cells of particular interest (Unger, 1997; Bouakaz et al., 2016; Belling et al., 2020). This minimally invasive acoustic techniques can selectively and accurately deliver various drugs or genes to an organ by localizing ultrasound to the target lesion (Lakshmanan et al., 2014; Du et al., 2018). It is believed that the existence of microbubbles (MBs) in the surrounding liquid when applying ultrasound to cells is essential in obtaining maximum gene transfer efficiency. Recent studies have shown that bubble reagents such as ultrasound contrast agents plays an important role in therapeutic ultrasound applications (Li et al., 2020). Clinical cases have been reported where MBs were intravenously injected during focused ultrasound irradiation for the purpose of opening the blood brain barrier (Abrahao et al., 2019). Numerous research papers have shown that bubbles smaller than one 100th of a millimeter in diameter, increases permeabilization of various drugs through the cell membrane and into the cytoplasm (Chowdhury et al., 2020).

The phenomenon of growth and collapse of MBs under an ultrasonic field, is known as “acoustic cavitation” (Suslick, 1989; Leighton, 1997). The collapse of ultrasound-irradiated bubbles is thought to be the underlying mechanism that cause transient pores in the cell membrane through which high velocity micro jet flow allows extracellular drugs or genes to penetrate living cells. (Greenleaf et al., 1998; Tachibana et al., 1999; Moosavi Nejad et al., 2011; Song et al., 2019; Kooiman et al., 2020). A new approach of using nanobubbles (NBs) instead of MBs have attracted attention as an alternative means for ultrasound-mediated gene therapy (Zullino et al., 2018). Nanobubbles, officially termed as ultra-fine bubbles (ISO, 2017), are defined as sub-micron diameter bubbles. Due to technical difficulty in identifying and observing sub-micro size bubbles, it was not until recently that NBs proved to really exist. Direct evidence for the existence of NBs in seawater was first reported in 1981 by Johnson and Cook (Johnson and Cooke, 1981). Since then, many researchers have investigated the existence, origin or physical and chemical properties of NBs (Hernandez et al., 2019). It has been found that NBs have several unique physical characteristic properties such as negligibly low buoyancy, negatively charged surface, radical formation and self-pressurization (Alheshibri et al., 2016; Sun et al., 2016; Yasui et al., 2018).

One of the reasons that sub-micro sized bubbles maybe more advantageous for gene therapy than MBs is the fact that NBs may potentially extravasate through the endothelial cell layer of the blood vessel, thus increasing NB accumulation in normal tissue and tumor vasculature, resulting in higher gene transfer efficiency rate (Yin et al., 2012; Wu et al., 2019; Pellow et al., 2020). Furthermore, it would be ideal if NBs could reduce irreversible cell damage induced by acoustic cavitation and at the same time deliver adequate quantities of genes into the cell plasma. Although acoustic cavitation can be triggered using various ultrasound parameters, it can be hypothesized that bubble size is one of the crucial factors among many others that are involved in the event of ultrasound-mediated gene therapy. Numerous studies have been conducted in optimization of MB size (Liao et al., 2014; Song et al., 2017; McMahon et al., 2020), however, as of today, no reports have yet been published on the influence of sub-micron sized bubble distribution for sonoporation-induced gene transfer. Here we demonstrated ultrasound mediated gene transfer with different NB size distributions *in vitro* with the aim of optimizing NBs for future therapies and understanding in more depth the mechanism of sonoporation in the sub-micro scale.

## Material and Methods

### Preparation of Nanobubbles

Human serum albumin-based NBs were prepared according to previous studies reported elsewhere (Lafond et al., 2018; Watanabe et al., 2019; Kida et al., 2020). Briefly, the air in a plastic container tube (height: 30 mm, outer diameter: 25 mm) was replaced with 15 ml of perfluoropropane gas (C_3_F_8_; Takachiho Chemical Industrial, Tokyo, JP) using a 23-gauge needle inserted through a small opening in a custom made cap. Ten-mL sterile solution of 0.06% human serum albumin (Albuminar-25; CSL Behring LLC, IL, United States) in opti-MEM (Thermo Fisher Scientific, Waltham, MA, United States) was added into the gas filled container tube. The C_3_F_8_ gas and albumin solution in the container were tightly sealed to prevent any liquid and gas leakage. The container tubes were then placed into a high-speed shaking-type tissue homogenizer device (Precellys Evolution; Bertin Instruments, FR) and shaken four times under the following conditions: 6,500 rpm, 60 s duration and 5 min pause on ice between each shaking phases. To extract uniformly sized NBs from the agitated suspension, centrifugation was carried out at 100 G for 10 min (MX-301; TOMY, Tokyo, JP). After removing the upper foamy-layer, the lower liquid-layer which included NBs was extracted from the test tube. The residual suspension was mixed evenly with pipetting and stored in 4°C until use for later described centrifugation, sonication or gene transfection experiments within 3 h.

### Centrifuge Treatment to Nanobubbles

The experiments were conducted to determine how NBs size distribution would under centrifugal force. The suspension containing NBs was transferred to a 15 ml conical tube (Greiner Bio-One, Oberösterreich, AT) and centrifuged at gravity acceleration 1000 G or 5000 G for 10 min (MX-301; TOMY, Tokyo, JP). NBs immediately after centrifugation were used for gene transfer experiments.

### Sonication Treatment to Nanobubbles

**FIGURE 1:**
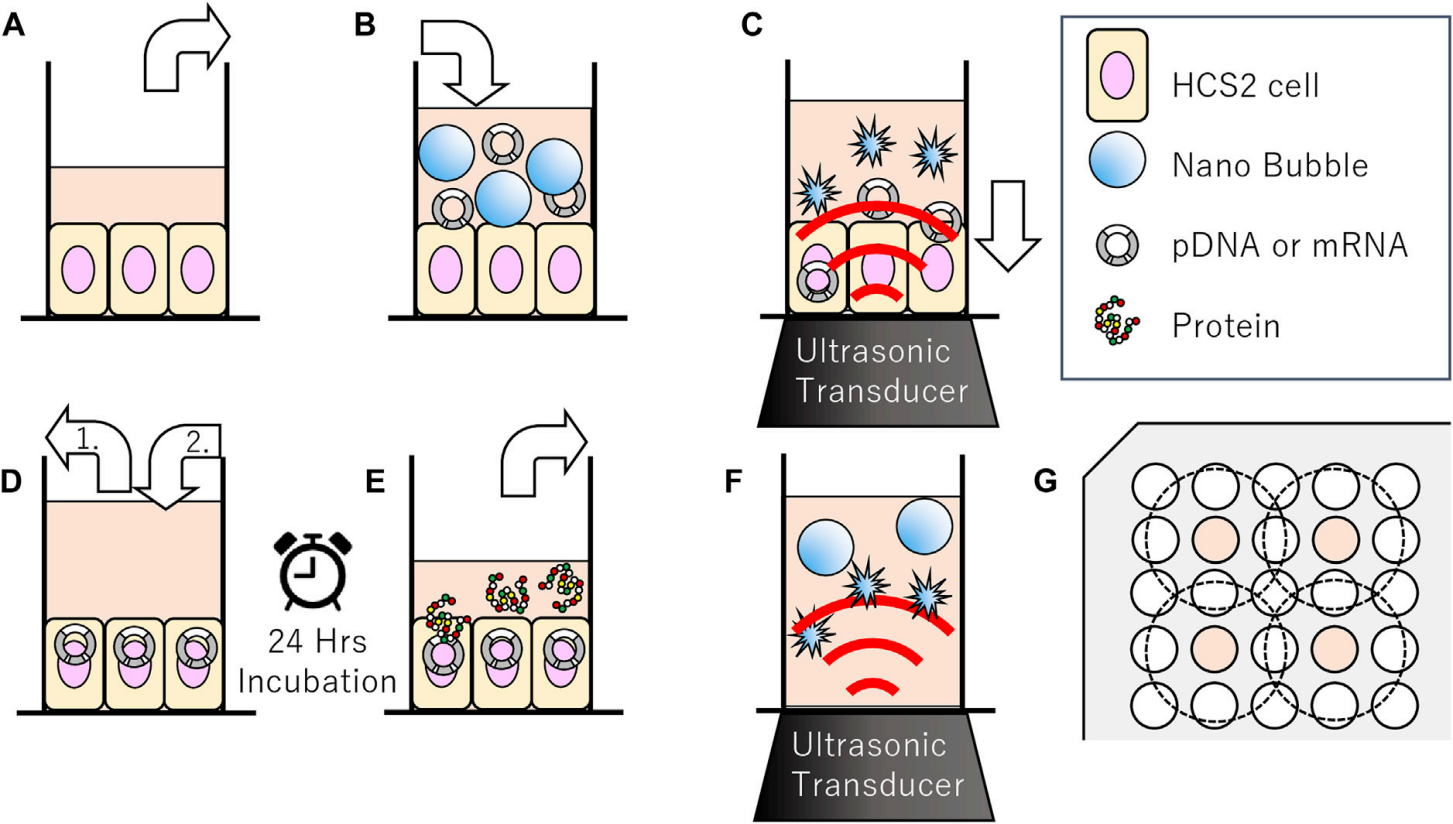
Schematic representation of methods of sonoporation or sonication treatment to nanobubbles in 96-well plates. **(A–E)** Methods of sonoporation. **(A)** Remove incubation medium from well of 96 multi well plate seeded with HSC-2 cells. **(B)** Fill wells with medium nanobubbles included. **(C)** Gene transfection by ultrasonic irradiation. (D-1) Aspiration of sonicated medium (D-2) Addition new incubation medium. **(E)** After 24 h incubation, collect supernatant for reporter assay. **(F)** Methods of sonication treatment to nanobubbles. Irradiation ultrasound to medium including nanobubbles. **(G)** Arrangement of wells seeded cells (indicated with color) and ultrasonic irradiation area (inside of dashed circle) on 96 multi-well plates.

In order to evaluate the affect of ultrasound to the NBs, size distribution of NBs was measured before and after sonication. NB suspension (100 μl) was placed within the acoustically transparent film based 96 multi-well cell culture plate (Sarstedt, Nümbrecht, NRW, DE). The culture plate was fixed above the surface of the ultrasound transducer via acoustic transmission gel (Aquasonic 100 gel; Parker lab, NJ, United States). The ultrasound was irradiated with a sonoporater (SP100; Sonidel Limited, Dublin, IRE) with transducer (diameter 1.6 cm), driving frequency of 1 MHz, burst rate 100 Hz and duty ratio of 50% (Figure 1A). The ultrasound irradiation method is identical to the later described micro scale *in-vitro* sonoporation system using 96 multi-wells plate which includes culture cells. The diameter of NBs was examined after sonication at various intensities (0, 2.5, 5.0 W/cm^2^) for a duration of 0, 5, 10, 20, 30, 45, 60 s.

### Physical Characterization of Nanobubbles

The physical character of NBs was measured as described previously (Watanabe et al., 2019; Kida et al., 2020). Briefly, the particle size of NBs was measured by nanoparticle tracking analysis (NTA) device (NanoSight LM10; Malvern Instruments, Worcestershire, United Kingdom). The nanoparticle suspension was illuminated by a 638 nm wavelength red laser. The nanoparticle movement was visualized by light scattering and the Brownian motion recorded by a CCD camera (C11440-50B; Hamamatsu Photonics K.K., Shizuoka, JP). The above system automatically detects the center position of nanoparticles and tracks each particle motion in a two-dimensional plane for later calculation of the average moving distance under Brownian motion. The image of particle movement with NTA was recorded for 60 s at room temperature. The range of particle size measurement of NTA method was adjusted from 10 to 1,000 nm. The particle size was estimated by the average moving distance to the Stokes-Einstein equation. The NBs suspension of 0.5 ml was injected into the sample measurement chamber of the Nanosight system with a 1.0 ml volume plastic syringe (Terumo, Tokyo, JP). Sample image capturing and data analysis were performed using the measurement application software (NTA 3.2 Dev Build 3.2.16). All sample measurement were performed independently for each sample. Particle size was presented as a mean and mode ±standard error of the average of three measurements. NBs size after centrifugation or ultrasound irradiation were compared with untreated samples.

**FIGURE 2:**
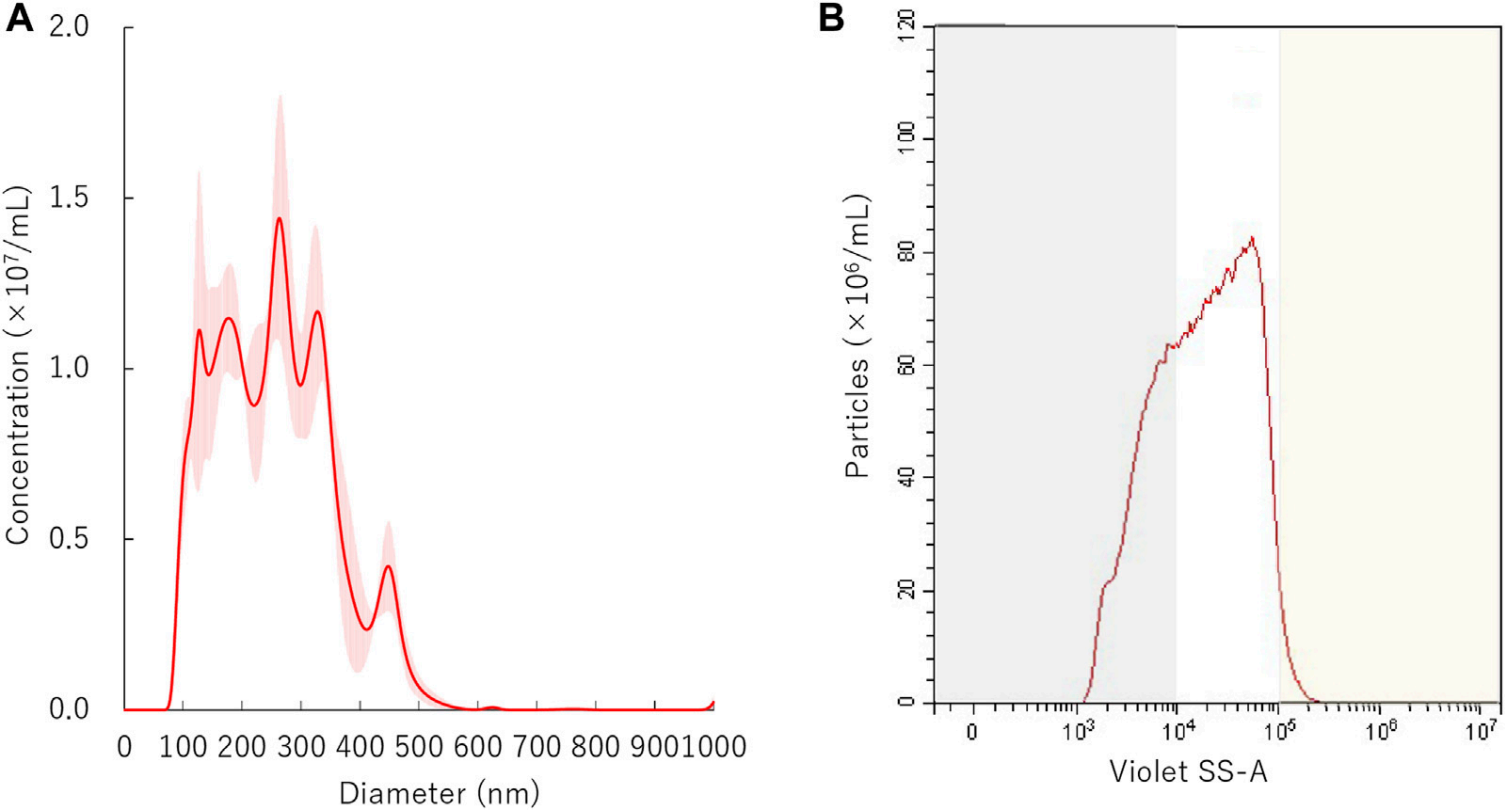
Distribution of nanobubbles. **(A)** Size distribution of nanobubbles measured by NTA. **(B)** The concentrations and distribution of nanobubbles by FCM. Gray or yellow shaded area indicates nanobubbles size less than 200 nm or more than 1,000 nm, respectively.

**FIGURE 3:**
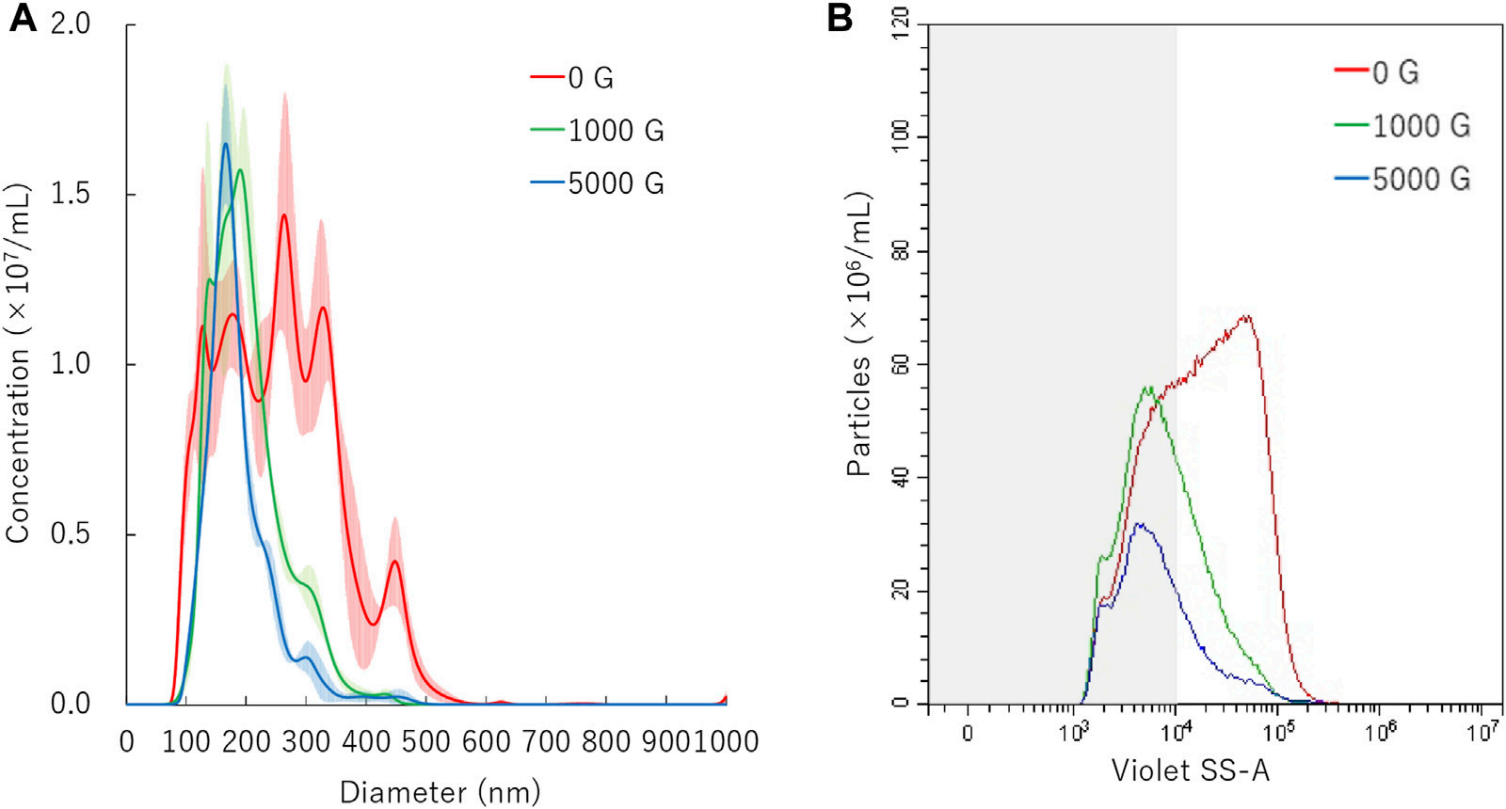
Nanobubbles size distribution after centrifugation. **(A)** Size distribution of nanobubbles (NTA) with or without centrifugation. **(B)** The concentrations and size distribution of nanobubbles with or without centrifugation (FCM). Gray shaded area indicates nanobubbles size less 200 nm.

**FIGURE 4:**
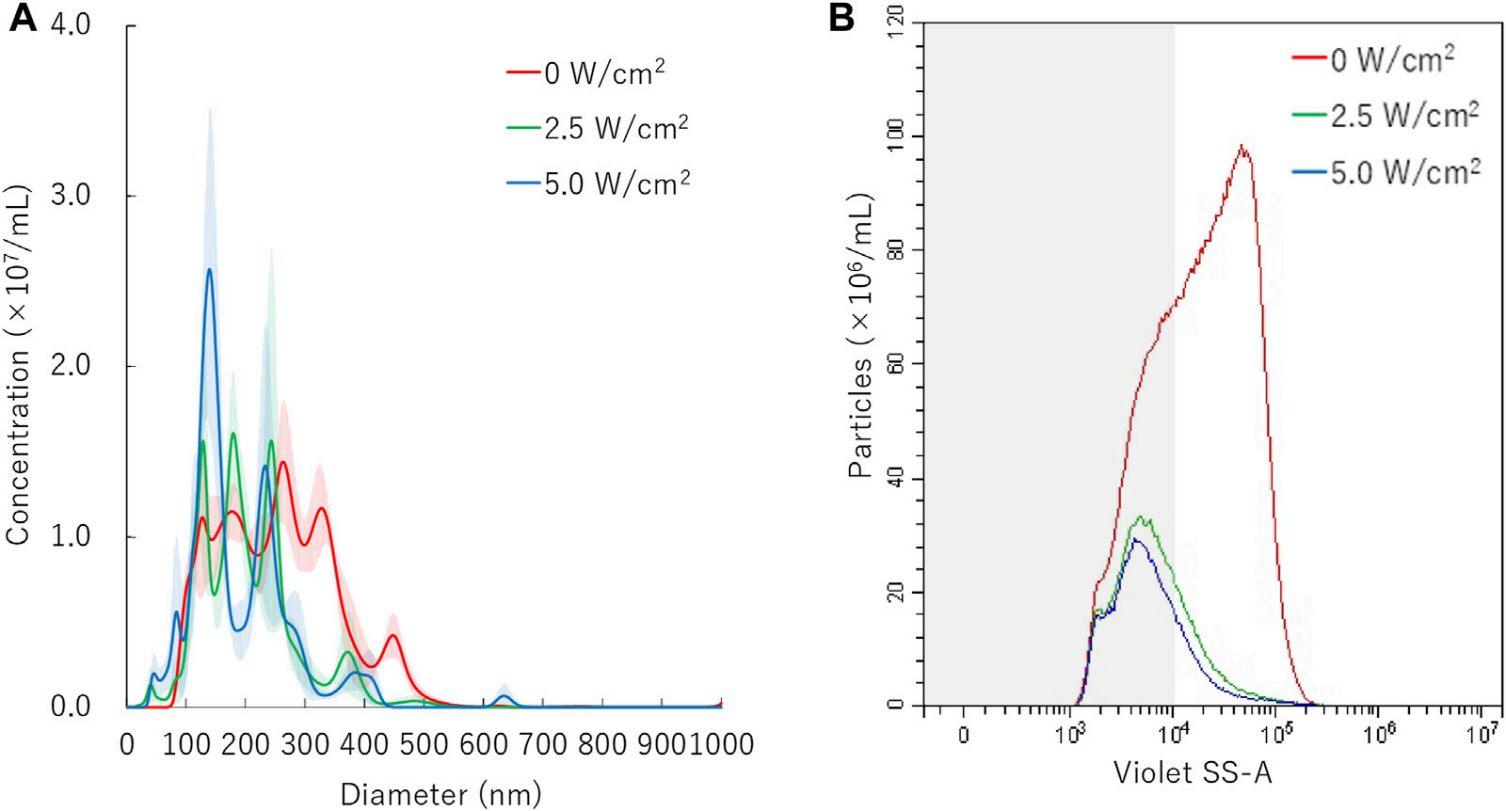
Nanobubbles size distribution with various sonication intensities. **(A)** Size distribution of nanobubbles (NTA) with or without sonication. **(B)** The concentrations and size distribution of nanobubbles with or without sonication (FCM). Gray shaded area indicates nanobubbles size less 200 nm.

**FIGURE 5:**
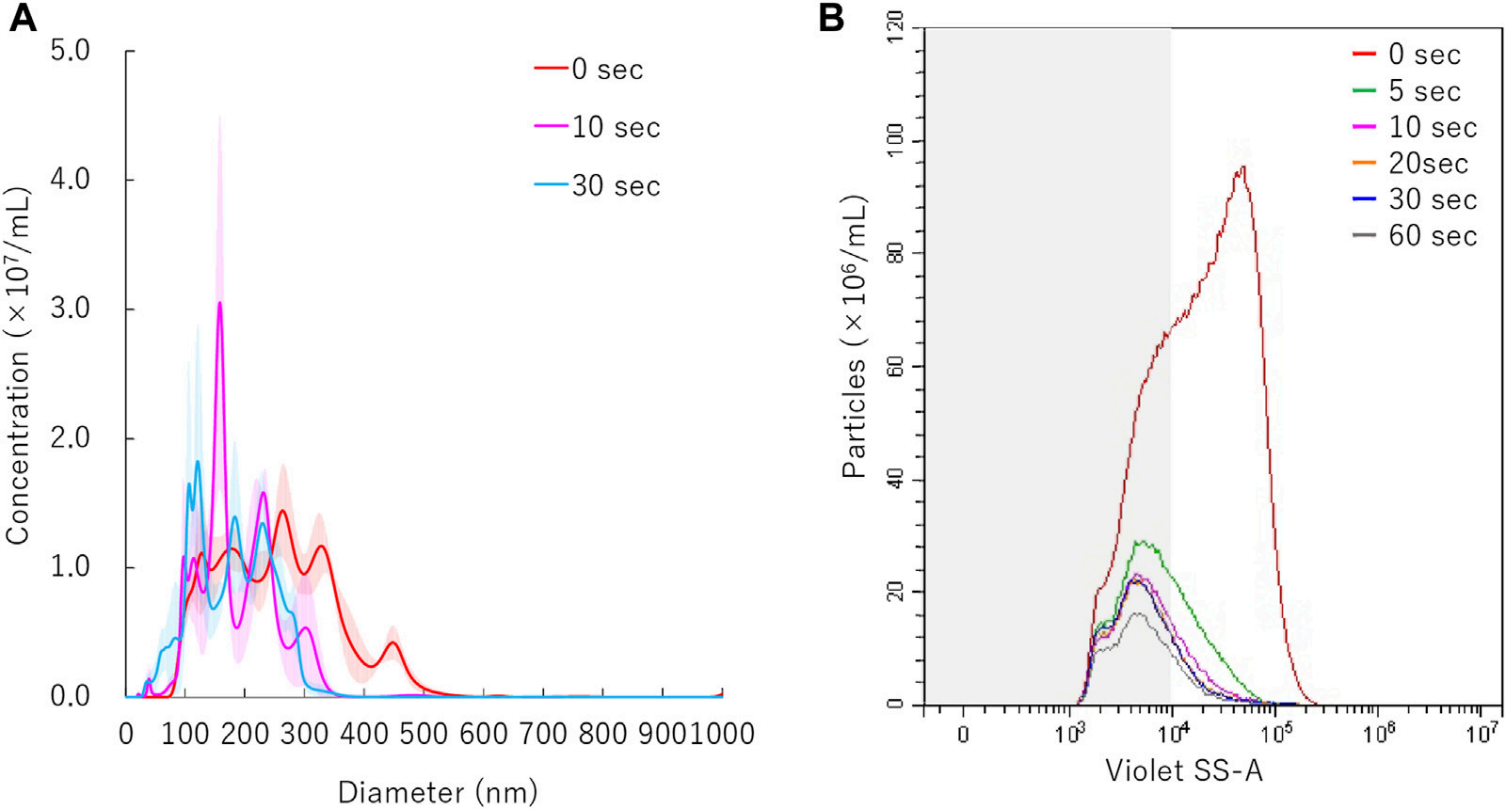
Nanobubbles size distribution with various sonication duration. **(A)** Size distribution of nanobubbles (NTA) with or without sonication. **(B)** The concentrations and size distribution of nanobubbles with or without sonication (FCM). Gray shaded area indicates nanobubbles size less 200 nm.

**FIGURE 6:**
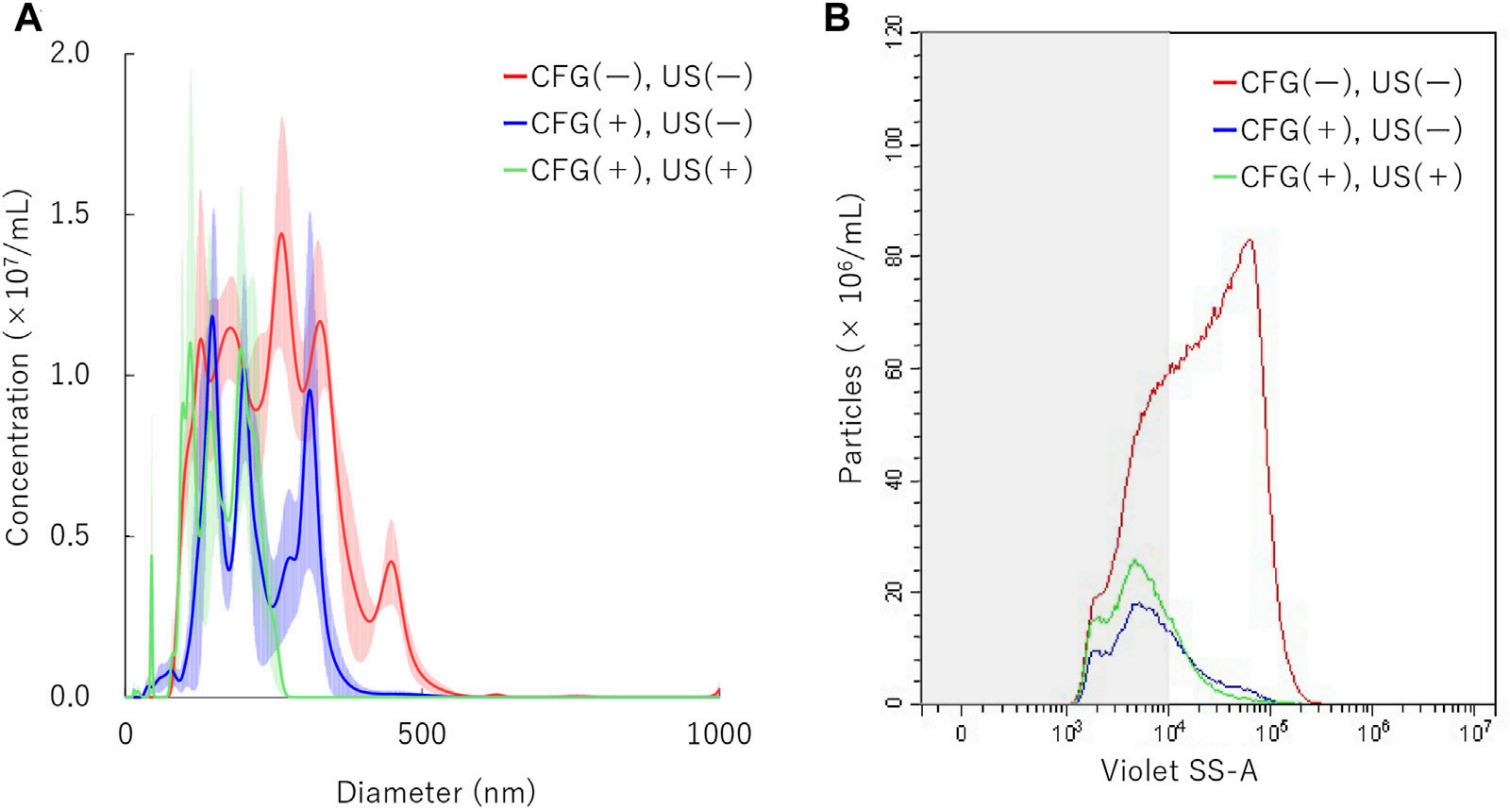
Nanobubbles size distribution by combination of centrifugation and sonication. **(A)** Size distribution of nanobubbles (NTA) with or without centrifugation and sonication **(B)** The concentrations and distribution of nanobubbles with or without centrifugation and sonication (FCM). CFG, centrifugation. US, ultrasound irradiation. Gray shaded area indicates nanobubbles size less 200 nm.

The size proportion and number of NBs were measured by a flow cytometer (CytoFLEX; Beckman Coulter, CA, United States). The flow cytometer was equipped with a 405 nm (violet) laser to detect the nanoparticles. The flow cytometer was set up to measure the Side Scatter (SS) from the violet laser for enhanced nanoparticle detection (Violet-SS). The Violet-SS signal resolution limitation for particle detection was 200 nm. Superior resolution can be obtained with SS than the Forward Scatter (FS) signal and is suitable for measurement of small particles (e.g., nanoscale particles). In order to relate Violet Side Scatter Area (SS-A) to a particle size, we calibrated the flow cytometer with beads of known size (Wisgrill et al., 2016; Zucker et al., 2016). The polystyrene standard beads (200 nm; qNano Calibration Particles; Izon Science, Christchurch, NZ, 500 nm; Archimedes Standard polystyrene beads; Malvern Instruments, Worcestershire, United Kingdom) were suspended in ultrapure water and measured beforehand with the flow cytometer. The acquired Violet SS-A signals of NBs were then analyzed by CytExpert analysis software version 2.0 (Beckman Coulter, CA, United States). A gate was created based on the size of standard beads in the range 200–500 nm for determining the size of our fabricated NBs. Before the experiments, the Violet SSC-A value of the 200 and 500 nm standard beads were over 10^4^ and over 10^5^, respectively. Using these data, the signal bands in which particles below 200 nm or over 500 nm exist were overlaid as gray or yellow shading area on the distribution graph of NBs (Figures 2–6). The proportion of concentration of NBs of 200 nm or more (200NB≤) was calculated by subtracting the concentration of NBs less than 200 nm (<200NB) from the total particle concentration of 100%. The number of particles of NBs were diluted 10-fold before measurement, and the concentration of the stock suspension were calculated, retrospectively. The NBs after centrifugation and ultrasonic irradiation were compared with the untreated NBs obtained before each experiment.

### Theoretical Calculation of Bubble Gas Volume

The theoretical gas volume of bubbles was calculated based on the result data of NTA in the equation below with reference previous study (Abenojar et al., 2020):

$$V=\frac{4}{3}\pi {\left(\frac{d-30}{2}\right)}^{3}\times \left(1\times 10^{-15}\right)$$

where ***V*** (nl) is theoretical gas volume of a single bubble, ***d*** (nm) is the mean diameter of the bubble. In this study, the shell thickness was assumed to 15 nm based on data from Albunex, which was commercially albumin-shell bubble contrast agents (Christiansen et al., 1994). Total gas volume per liquid volume (nl/ml) of each sample was obtained by multiplying the volume per bubble with the concentration on NBs on data of FCM.

### Cell Culture

Oral squamous carcinoma cell line HSC-2 was purchased from JCRB (Japanese Cancer Research Bank) cell bank and cultured in Minimum Essential Medium (MEM; Nacalai Tesque, Kyoto, JP) with 10% Fetal Bovine Serum (In Vitrogen, Tokyo, JP). Cells were maintained at 37.0°C in humidified air with 5% CO_2_. HSC-2 cells were collected by trypsin-EDTA (Gibco, NY, United States). They were then washed and maintained in fresh medium immediately before each sonoporation experiments. On the same day before the experiments, cells were collected and centrifuged at 100 G for 5 min. They were seeded 2 × 10^4^/well to the lummox 96 multi-well black plate, every other row and column, in order to prevent interaction of nearby ultrasound irradiation to each other (Figure 1F). The cell line was free of viral pathogens with initial viability of more than 99% before use in the actual experiments.

### Preparation of pDNA and mRNA

pNL1.3CMV [secNluc/CMV] encoding secreted NanoLuc (secNluc) luciferase was purchased from Promega (Madison, WI, United States). It was constructed as previously mentioned (Kida et al., 2020). pDNA was amplified in *Escherichia coli* strain DH5α. After isolation, pDNA was purified using endotoxin-free plasmid purification kit. The pDNA was dissolved in Milli-Q water and stored at −20°C prior to each experiment.

Gluc mRNA was constructed as previously mentioned (Oyama et al., 2021). DNA templates for *in vitro* transcription (IVT) of mRNA were constructed by inserting a protein-expressing fragment into a pSP73 vector (Promega, Madison, WI, United States) that included a T7 promoter. Prior to the insertion, a 120-bp poly A/T sequence was cloned into the pSP73 vector downstream of the protein-coding sequence, so that mRNA possessing a 120 adenine poly(A) tail at the 30 terminal end could be obtained by a simple procedure of IVT from the pSP73-poly(A) vector. The protein-expressing fragments were obtained from DNAs encoding firefly luciferase (pGL4; Promega, Madison, WI, United States).

### Micro Scale *In-Vitro* Sonoporation System Using 96 Multi-Wells Plate

pDNA encoding secNluc or mRNA encoding Gluc were respectively added to NBs at 10 μg/ml and used for *in vitro* sonoporation treatment. The schematic representation of all steps of the experiments are described in Figure 1. Each HSC-2 cell culture medium of the 96 multi-well plate with an acoustically transparent bottom were replaced with 100 μl NBs medium which including 1,000 ng pDNA or mRNA (Figures 1A,B). Ultrasound (SP100, Sonidel Limited, Dublin, IRE) was irradiated to the culture plate bottom containing HSC-2 cells, NBs and genes (Figure 1C). After ultrasound irradiation treatment, the suspension containing NBs were removed. Then 100 μl of culture medium was re-filled to each culture well and incubated at 37°C in a humidified, 5% CO_2_ atmosphere (Figure 1D). After 24 h, luciferase expression assay and cell viability assay were performed by the method described below in Figure 1E.

The gene transfer efficiency was examined at various condition of NBs treated with various centrifugal forces (0, 1,000, 5000 G) or ultrasound irradiation with various intensities (0, 2.5, 5.0 W/cm^2^) for 30 s. Each of the sonication time-dependent efficiency of gene transfer were investigated in the presence or absence of NBs.

### Evaluation of Luciferase Expression

*In vitro* luciferase activity was determined by bis-Coelenterazine (bis-CTZ) assay kit (JNC, Tokyo, JP) using Spark Multimode Microplate Reader (Tecan, Männedorf, Zürich, CH). After 24 h incubation after cell sonication, 10 μl of culture supernatant were retrieved from each incubation well on Costar 96 well white solid plate (Corning, NY, United States). Relative luminescence unit (RLU) value was plotted for 1 μg/100 μl bis-CTZ solution.

### Cell Viability Assay

The number of viable HSC-2 cells was measured by colorimetric method using 3-(4,5-dimethylthiazol-2-yl)-5-(3-carboxymethoxyphenyl)-2-(4-sulfophenyl)-2H-tetrazolium (MTS) to determine the number of viable cells in cytotoxicity assay [CellTiter 96 AQueous One Solution Cell Proliferation Assay system (Promega, Promega, Madison, WI, United States)]. 20 μl of Cell Titer Solution Reagent was added to each well where a part of the supernatant was removed for the luciferase assay. After 2 h incubation, the absorbance was recorded at 490 nm using a 96-well plate reader Multiskan Go (Thermo Fisher Scientific, MA, United States). The survival rate of treated cells was calculated as the ratio of the number of surviving cells to the number of control non-treated surviving cells.

### Statistical Analysis

Measurement data were displayed as mean ± standard error of the mean (s.e.m). Data was analyzed using one-way ANOVA with Tukey’s multiple comparisons post-test or unpaired *t*-test including Welch’s correction. The statistically significant differences between various groups were analyzed using EZR 1.54 (Saitama Medical Center, Jichi Medical University, Saitama, Japan) (Kanda, 2013). The probability value of *p* value <0.05 was considered statistically significant.

## Results

### NBs Size Distribution

The initial size distribution of the NBs before centrifuge is shown in Figure 2A. The mean size of NBs was 254.7 ± 3.8 nm and mode 219.1 ± 46.5 nm. The overlaid Violet-SS signal intensity histogram of NBs are shown in Figure 2B. The total number of NBs before centrifuged or sonicated NBs was approximately 9.7 × 10^8^/ml. The number of NBs with a diameter of 200 nm or more (200NB≤) accounted for 66.9% of the total number of NBs, and particles smaller than diameter 200 nm (<200NB) was 33.1%.

### NBs Size Distribution After Centrifugation

The mean diameter of NBs after 10 min centrifugation treatment was 201.9 ± 0.4 nm or 187.3 ± 4.8 nm on NTA measurement after centrifugal acceleration of 1000 G or 5000 G, respectively (Figure 3A). The FCM measurement resulted in a total number of NB decrease from 8.6 × 10^8^/ml to 4.8 × 10^8^/ml (56.3%) or 2.6 × 10^8^/ml (29.9%) of after 1000 G or 5000 G centrifuge treatment, respectively. The proportion of 200NB ≤ decreased from 67.9% to 36.6% (1000 G) or to 30.3% (5000 G), while the proportion of <200NB increased from 32.1% to 63.4% (1000 G) or to 69.7% (5000 G) (Figure 3B). Results showed that 200NB≤ was more likely to be lost by centrifugation than <200NB. Most of the 200NB≤ were lost, while those of <200NB were retained even when the centrifugal force was increased to 1000 G or more. The retention rate of <200NB was 111.2% and 65.1% after centrifuge treatment of 1000 G, 5000 G, respectively, whereas a large proportion of 200NB≤ were lost or destructed with centrifuge treatment of 1000 G or 5000 G. The alteration of NBs character (mean size, NB concentration) after centrifugation were summarized in Supplementary Table S1. Total gas volume was reduced from 5.11 nl/ml to 1.28 nL/ml or 0.53 nl/ml with centrifuge treatment of 1000 G or 5000 G, respectively.

### NBs Size Distribution After Sonication

Results of NTA are shown in Figure 4A. Sonication to NBs suspension with ultrasound of intensity of 2.5 W/cm^2^ or 5.0 W/cm^2^ for 30 s resulted in the mean size of NB decrease from mean 254.7 ± 3.8 nm to mean 203.8 ± 11.3 nm or mean 191.4 ± 14.3 nm, respectively. FCM resulted in total number of NBs decrease from 1.1 × 10^9^/ml to 2.6 × 10^8^/ml (24.0%) or 2.1 × 10^8^/ml (19.6%), after 2.5 W/cm^2^ or 5.0 W/cm^2^ sonication, respectively. FCM measurement showed the proportion of 200NB ≤ decrease from 69.8% to 29.7% or 25.2%, respectively (Figure 4B). 200NB≤ was 2.0 or 1.8 fold more less than <200NB after 2.5 or 5.0 W/cm^2^ sonication, respectively. The alteration of NBs character (mean size, NB concentration) with various sonication intensities were summarized in Supplementary Table S2. Total gas volume was reduced from 6.53 nl/ml to 0.71 nl/ml or 0.53 nl/ml after 2.5 or 5.0 W/cm^2^ sonication, respectively.

The mean particle size of NBs irradiated with 5.0 W/cm^2^ intensity ultrasound, decreased from before sonication size mean from 254.7 ± 3.8 nm to 186 ± 9.8 nm within the first 10 s (Figure 5A). The size of NBs irradiated for 30 s had a mean of 178.9 ± 5.4 nm, which was almost the same as that of NBs irradiated for 10 s. Total number of NBs decreased to from 1.0 × 10^9^/ml to 2.5 × 10^8^/ml (24.0%) after 5 s sonication, and eventually reached to 1.2 × 10^8^/ml (11.4%) after 60 s. The proportion of 200NB ≤ decreased to 35.3% or 21.9% from 69.6% with 5 s or 60 s sonication, respectively (Figure 5B). These experiments revealed that 200NB≤ were mostly lost or destructed by early period of irradiation of moderate intensity ultrasound. On the other hand, it was found that <200NBs is difficult to destroy even if it is irradiated with a high intensity sonication for long durations. The alteration of NBs character (mean size, NB concentration) with various sonication duration is summarized in Supplementary Table S3. Total gas volume was reduced from 5.94 nl/ml to 0.36 nl/ml or 0.28 nl/ml after 10 s or 30 s sonication, respectively.

### NBs Treated With Centrifugation and Sonication

To determine the physical behavior of NBs in identical condition as main gene transfer experiments, additional measurements were conducted both under centrifugation and sonication treatment. NBs were centrifuged at 5000 G for 10 min and then sonicated at 5.0 W/cm^2^ for 30 s. Results showed that NB particle size was reduced to 217.1 ± 8.0 in the first centrifugation. It was reduced to 163.9 ± 4.6 nm in the following sonication (Figure 6A). Measured with FCM showed total number of NBs alteration from 9.4 × 10^8^/ml to 1.5 × 10^8^/ml after first centrifugation, then to 1.9 ×10^8^/ml (20.2%) after sonication (Figure 6B). The proportion rate of 200NB ≤ has decreased from 70.6% to first 35.3% with centrifugation, then to 25.4% with sonication. The alteration of NBs character (mean size, NB concentration) with combination of centrifugation and sonication were summarized in Supplementary Table S4. Total gas volume was reduced from 5.58 nl/ml to 0.24 nl/ml by combination with centrifugation and sonication.

### Effect of NBs Size on Gene Transfer Efficiency

**FIGURE 7:**
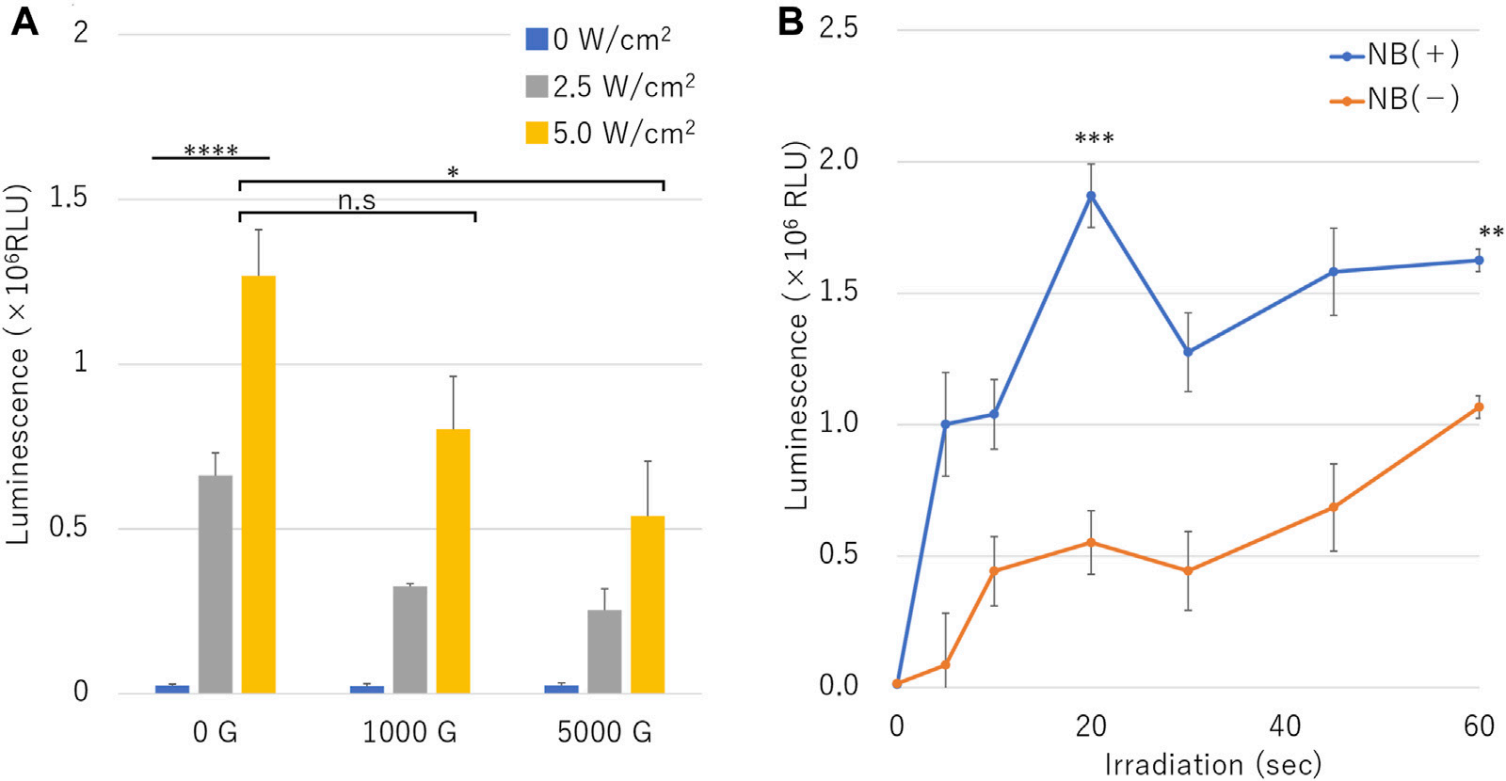
pDNA transfection efficiency by sonoporation using different sized nanobubbles or irradiation time. **(A)** Expression of secreted luciferase protein by sonoporation using combinations of centrifuged bubbles and ultrasound. **(B)** Ultrasonic irradiation time dependent profile of luciferase expression in conditions with or without nanobubbles. (RLU, relative luminescence units). The data are presented as the mean ± standard error of the mean (s.e.m.). Statistical significance was assessed **(A)** one-way ANOVA with Tukey’s multiple comparisons post-test or **(B)** unpaired *t*-test including Welch’s correction (*p < 0.05, ***p < 0.001, ****p < 0.0001, n. s not significant) (*N* = 3).

Figure 7 shows the luciferase assay in microscale *in-vitro* sonoporation of pDNA of the NBs treated with different centrifugal forces and different ultrasound intensities, relative luminescence unit (RLU) value increased in proportion accordingly to acoustic intensity under all conditions with or without NB centrifugation treatment (Figure 7A). RLU value at combination of the uncentrifuged NBs (0 G) and 2.5 or 5.0 W/cm^2^ ultrasound irradiation was 0.66 ± 0.07 (10^6^) or 1.27 ± 0.14 (10^6^), which was 26.8 or 51.4 folds that of cells without sonication (*p* = 0.0006). The RLU value results of centrifuged NBs at 1000 G or 5000 G and 5.0 W/cm^2^ ultrasound irradiation was 0.80 ± 0.16 (10^6^) or 0.53 ± 0.17 (10^6^), respectively, which was 0.6 or 0.4 times that of uncentrifuged NBs (*p* = 0.07416, *p* = 0.025954).

In the assay result of pDNA transfection efficiency over duration of sonoporation with or without NBs is shown in Figure 7B. RLU value under the condition without NBs increased to 1.07 ± 0.09 (10^6^) in 60 s sonication. RLU value under the condition of NBs without centrifugation increased to 1.00 ± 0.19 (10^6^) in 5 s sonication. RLU value was 1.87 ± 0.12 (10^6^) in 20 s, which was almost 3.4 folds compared to that of cells without the presence of NBs (Figure 7B) (*p* = 0.002686).

**FIGURE 8:**
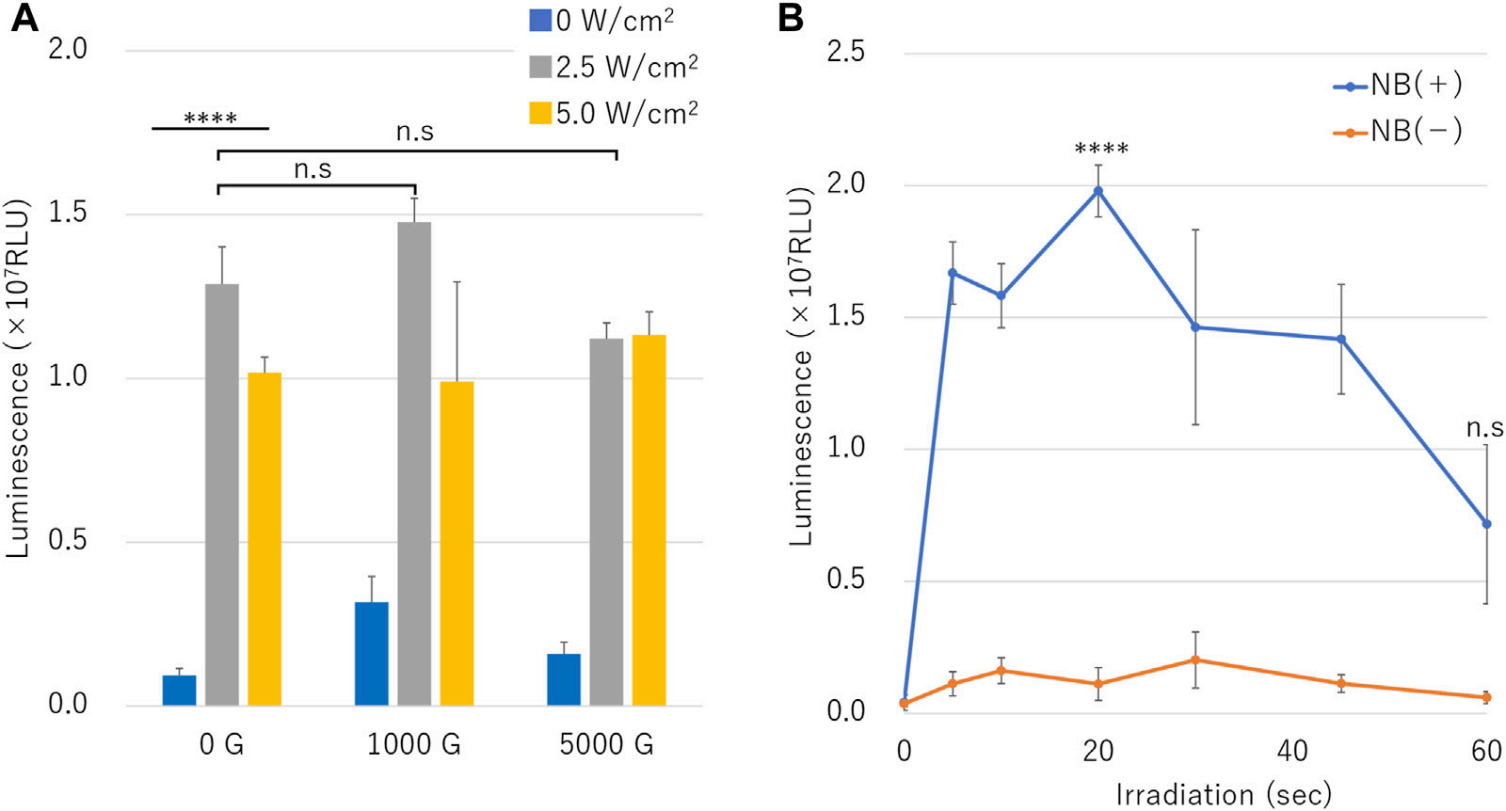
mRNA transfection efficiency by sonoporation using different sized nanobubbles or irradiation time. **(A)** Expression of secreted luciferase protein by sonoporation using centrifuged bubbles and ultrasound. **(B)** Luciferase expression in conditions with or without nanobubbles. (RLU, relative luminescence units). The data are presented as the mean ± standard error of the mean (s.e.m.). Statistical significance was assessed **(A)** one-way ANOVA with Tukey’s multiple comparisons post-test or **(B)** unpaired *t*-test including Welch’s correction (****p < 0.0001, n. s not significant) (*N* = 3).

Luciferase assay results of naked mRNA transfection is shown in Figure 8. Treatment with various ultrasound intensities in the presence of different centrifuged NB resulted in highest luminescence with NBs centrifuged at 1000 G and 2.5 W/cm^2^ ultrasound irradiation (RLU value: 1.48 ± 0.07 (10^7^)) (Figure 8A).

Cells irradiated with ultrasound intensity of 2.5 W/cm^2^ in the presence of NB centrifuged at 1000 G or 0 G showed higher RLU value than cells irradiated with 5.0 W/cm^2^. RLU value cells treated in presence of uncentrifuged NBs (0 G) and 2.5 or 5.0 W/cm^2^ ultrasound irradiation was 1.29 ± 0.11 (10^7^), 1.02 ± 0.05 (10^7^), respectively (*p* = 0.000177), which was 13.7 or 10.8 fold that of cells untreated by ultrasound. RLU value of cells sonicated with 5.0 W/cm^2^ compared to 2.5 W/cm^2^ was 0.8, 0.7 or 1.0 fold under the conditions of centrifuged NBs at 0 G, 1000 G, or 5000 G, respectively. The affect of RLU value of cells sonicated at 5.0 W/cm^2^ of ultrasound showed attenuation in the presence of centrifuged NBs at 1000 G or 5000 G (1.0 or 1.1 times compared to 0 G, *p* = 0.473,330, 0.163,062). mRNA transfection efficiency over the duration ultrasound intensities of 10 or 60 s with or without the presence of NBs showed luminescence significant increased to 0.16 ± 0.04 (10^7^) and significant decreased to 0.06 ± 0.02 (10^7^), respectively. RLU value under the condition of NBs without centrifugation showed 1.67 ± 0.12 (10^7^) in 5 s. RLU value increased to 1.98 ± 0.10 (10^7^) in 20 s and decreased to 0.72 ± 0.30 (10^7^), which was almost 17.9 or 12.1 times compared to of condition without NBs, respectively (Figure 8B) (*p* = 0.000096).

**FIGURE 9:**
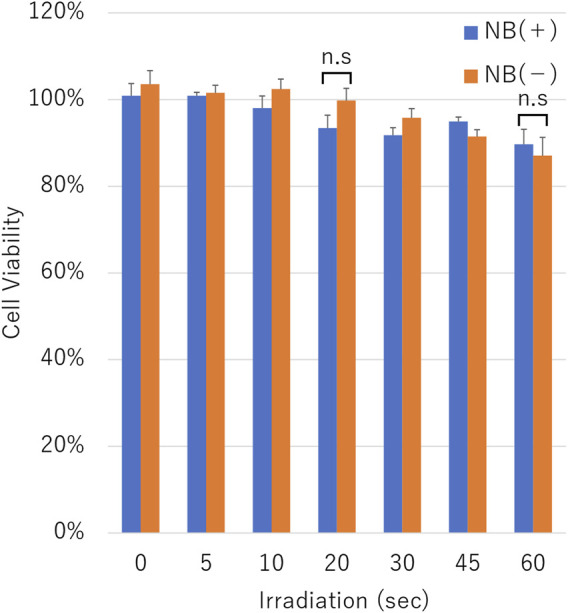
Cell viability after sonication. Cell viability after sonication with or without nanobubbles. The data are presented as the mean ± standard error of the mean (s.e.m.). Statistical significance was assessed unpaired *t*-test including Welch’s correction (n.s not significant.) (*N* = 3).

The cell viability assay was conducted on cells after pDNA transfection using uncentrifuged NBs. The cell viability was decreased in proportion to the duration of ultrasound irradiation in the condition with or without NBs. Cell viability with or without NBs for 60 s duration irradiation was 89.7 ± 3.5% or 87.1 ± 4.2%, respectively (*p* = 0.375,801) (Figure 9).

## Discussion

Improvement of gene delivery into cells and optimization of various transfection methods have become an important objective for scientists engaged in bench top gene therapy experiments as well as for clinicians. Among the many non-viral vector modalities investigated, combination of microbubbles (MBs) and ultrasound for intracellular gene delivery has shown to be one of the most promising. However, despite of these great expectations, several limitations still remain which includes relatively low gene transfer efficiency compared to viral-vector gene therapy modalities. Thus, it is necessary to explore the various parameters related to ultrasound mediated gene therapy that would ultimately lead to increased gene transfer for future therapeutic application in the clinical setting.

To date, a large number of experiments on ultrasound mediated gene transfer using MBs have been conducted on various cells and in organs (Endo-Takahashi and Negishi, 2020; Walsh et al., 2021). Most of the bubbles evaluated were originally developed as an ultrasound contrast imaging agent thus requires physically stable characteristics in order to achieve high echogenic acoustic signals. For this reason, a large portion of the bubbles investigated have a stabilized hard shell composed with biocompatible phospholipids (Greenleaf et al., 1998; Fix et al., 2015). Reports have shown that the outer shell can be chemically modified with various antibodies (Wu et al., 2016; Song et al., 2018; Hamano et al., 2019), peptides (Xie et al., 2016a; Xie et al., 2016b; Jing et al., 2016; Hamano et al., 2019), pDNA (Song et al., 2018), mRNA (De Temmerman et al., 2011; Dewitte et al., 2014; Dewitte et al., 2015) or siRNA (Xie et al., 2016a; Jing et al., 2016; Wu et al., 2016). These modifications have been successful in increasing targetability to cancer cells thus offering substantial outcome over non-targeted bubbles.

Furthermore, the lipid bilayer membrane that composes the outer shell of the MBs are said to alter the acoustic cavitation threshold. Theoretical evaluations have shown that the molecular weight of polyethylene glycol (PEG) chemically-bonded to phospholipid shell of the bubbles affect the induction of inertial cavitation (Wrenn et al., 2012). It was concluded in this study that membrane phase behavior influences the kinetics and mechanisms of lipid-based MB for sonoporation. On the other hand, the lipid shell of bubbles is easily trapped by the reticuloendothelial system (RES) such as Kupffer cells of the liver *in vivo*, thus the bubble shell surface are required to be further modified with PEG to avoid this biological reaction (Yan et al., 2020). It is suggested that MBs that function as cavitation nuclei without lipid shell may help avoid trapping by RES and eventually improve retention in the specified tissue for gene transfer.

In our previous study, we developed a method for generating stabilized non-lipid sub-micron sized bubbles consisted of albumin material. Certain proteins, such as albumin, are inherently foamy. At the gas-liquid interface of bubbles, proteins that reach the interface expose the hydrophobic region of their molecules to the gas phase and are replaced by water molecules in a higher energy state. It has long been known that a conformational change of the protein molecule is triggered at this time and that it is positioned at the gas-liquid interface (Ter-Minassian-Saraga, 1981). It is not clear what the shell structure of our nanobubbles (NBs) is, but albumin may have a similar position at the gas-liquid interface. Nevertheless, our NBs have been well characterized and in addition to bubble size distribution measurements, cavitation-threshold and rheological parameters were evaluated (Lafond et al., 2018). Compared to lipid shell-based NBs, our albumin stabilized NBs revealed a broader size distribution range and exhibited more sensitivity to various ultrasound intensities and frequencies. We showed that the albumin NBs had lower inertial cavitation threshold than lipid shelled NBs in an *in vitro* cell suspended experiment setup. Results suggested that albumin NBs irradiated with 0.8–1.0 W/cm^2^ intensity ultrasound tended to easily collapse and enhanced cancer cell disruption by up to 6.8 fold compared to ultrasound alone (Watanabe et al., 2019). Furthermore, we demonstrated gene transfer into *in vitro* attached cell culture monolayers and in mice by albumin stabilized NBs in conjunction with a hand-held portable ultrasound imaging device (Kida et al., 2020). The efficiency of gene transfer by albumin NBs was directly shown to be influenced by its physical properties and acoustic sensitivity. Nevertheless, in this case, albumin NBs induced less damage to the cell membrane compared to lipid shelled NBs during ultrasound-mediated gene transfection. In the present study, overall, both plasmid DNA and messenger RNA gene transfer into cells were similarly observed in greater quantities in the presence of NBs. The results are in agreement with our previous observations the phenomenon that NBs play an important role in the impact of sonoporation. Previous other studies aimed at delivering mRNA to deeper organs of the body by sonoporation required mRNA to be loaded into carriers to avoid degradation by RNases (De Temmerman et al., 2011; Dewitte et al., 2014; Dewitte et al., 2015). This study demonstrated that carrier-free mRNA could be delivered by sonoporation in applications such as gene-based vaccination if delivered promptly to avoid RNase contact.

Few analyses have been conducted so far on the relationship between gene transfer efficiency and bubble size in the sub-micron scale. In the present study, we firstly compared the NB size distribution before and after ultrasound irradiation. It was found in our NTA and FCM measurements that while NB diameter greater than 200 nm (200NB≤) rapidly collapsed in the early phase of ultrasonic irradiation, the number of NBs smaller than 200 nm (<200NB) remained relatively unchanged, eventually reaching a plateau concentration level comparable to that of non-irradiated NBs within the same size range. This result can be explained with a mathematical model for generation and reduction of NBs by ultrasonic irradiation which was proposed and confirmed experimentally in a previous study (Yasuda et al., 2019). Although the viscosity of the non-pure solution used in our experiments was not measured, it can easily be estimated that the bubble diameter that resonates with the 1 MHz ultrasound frequency according to Minnaert resonances will be much larger than the NBs (Minnaert, 1933). If a resonance relationship exists between NBs and ultrasound frequency based on Minnaert resonances, higher frequency of ultrasound could be suitable for resonating NBs. A more optimal acoustic parameters for NBs would reduce the ultrasonic energy required for sonoporation. However, there is no evidence as of now that this hypothesis could directly be applied to our NBs. More analysis should be carried out by varying ultrasound frequency perhaps to higher range or lowering ultrasound intensity to the minimal limit.

On the other hand, the peak of sonicated <200NB exceeded that of untreated <200NB on some conditions of NTA. One can make the argument that ultrasound itself can produce NBs to some extent. Thus, it can be postulated that very small NBs can independently generate cavitation nucleus during ultrasound irradiation but are unable to resonate until they exceed a certain bubble size and do not collapse immediately. However, once the bubble size crosses the minimum diameter size borderline for acoustic resonance to occur, they will start to grow rapidly and later collapse. The same mechanism may have affected intracellular gene transfer efficiency in our experiments but of course, it is not clear exactly why the larger sized NBs contributed more to increase gene transfer. Further detail evaluation is required to elucidate the physics and acoustic cavitation mechanism relating to this phenomenon.

Secondly, our results showed that similarly to the NBs treated with ultrasound, relative centrifugal force caused loss of relatively larger bubbles. It has been reported previously that centrifugation reduces the diameter and concentration of NBs (Oeffinger and Wheatley, 2004; Zhang et al., 2019). However, the exact physical principle of this phenomenon has not been fully investigated. The terminal velocity for a small spherical particle including a bubble moving in a viscous liquid can be roughly described by the well-known Stokes’ law (Stokes, 1851; Takahashi, 2005).

$$V=1/18\times gd^{2}/v$$

where ***V*** (m/s) is the rising velocity of the bubble, ***g*** (m/s^2^) is the gravitational acceleration, ***d*** (m) is the diameter of the bubble, and ***ν*** (m^2^/s) is the kinematic viscosity of water. However, it has not yet been proven that the terminal velocity of NBs, which is much smaller than that of MBs, can be applied to this law (Lee and Kim, 2005; Takahashi, 2005). If this were the case, the terminal velocity of a 100 nm NB in pure water at 20°C would be calculated as 3.3 μm/10 min. Since the rate of ascent is proportional to the square of the bubble radius and the gravitational acceleration, it is in agreement that the present experiment results showed that larger NBs (200NB≤) is more likely to be lost during centrifugation than smaller NBs (<200NB) due to high relative centrifugal force. Alternatively, relative centrifugal force may have collapsed the bubbles and transform it to potential cavitation nucleus. This suggests that the loss of bubbles due to centrifugation may at first glance appear to be irreversible. On the other hand, under intense and long duration ultrasound irradiation conditions, there is a possibility that a number of bubbles could have revived again to a stabilized NB. Additional observation of the physical behavior of NBs under low intensity and short duration ultrasound may reveal the underlying mechanism related to bubble loss by centrifugation. Nevertheless, the centrifuge pre-treated NBs greatly affected the gene transfer efficiency thus suggesting that larger size NBs contributed more to induction of sonoporation. Alternatively, an imbalance of static pressure in the liquid due to centrifugal forces near the liquid surface could have caused a phenomenon similar to “centrifugal pump cavitation” (Stopa et al., 2014) in the bubbles. Again, as this hypothesis is based on limited information from our study, more experimentation is required to confirm the true physics of this phenomenon.

It was reported that the ability of MBs to transfer genes is proportional to the size of the bubbles (Liao et al., 2014). Our experimental results reveal that this trend also applies to NBs. The initial early collapse of 200NB ≤ coincided with the surge in gene transfer. It has also been reported that the efficiency of MB collapse appears to be conversely proportional to the MB size in their study (Liao et al., 2014). However, in our experiments, 200NB≤ was more likely to decay at a rapid velocity than <200NB. Therefore, indicating that sonoporation efficiency may not be determined solely by the diameter of the bubble. The early disruption of 200NB ≤ seemed to be associated with initial precipitous increasing of pDNA and mRNA transfer efficiency. In contrast, the cavitation bubble diameter immediately before collapse may be the determining factor which relates to the resonance bubble diameter (Leighton, 1997). Additionally, it is suggested that NBs size close to the resonance bubble diameter determined from the ultrasonic frequency, causes induction of cavitation thus leading to sonoporoation. The parameters such as ultrasound frequency and intensity were not fully optimized in the present study. It is necessary to perform analysis with different parameter to reduce the damage to cells or tissues, and to achieve highly efficient gene transfer.

Nevertheless, previous studies have shown that in opening the blood brain barrier (BBB) with MBs, the mechanical disruption induced by cavitation greatly depends on the MBs size (Song et al., 2017; McMahon et al., 2020). These studies suggest that not only the size of the bubbles, but also the total volume of gas contained in the liquid affects the gene delivery efficiency. Our study found that centrifugation between 0 and 5,000 g decreased the concentration of NBs, the percentage of 200NB≤, and the total gas volume in the solution. Using these pretreated NBs for sonoporation reduced both the efficiency of pDNA and mRNA transfection. While ultrasound irradiation of 5 W/cm^2^, 30 s to NB without centrifuge pretreatment lost more than 90% (0.46 nl/6.53 nl or 0.28nl/5.94 nl) of total gas volume. The volume lost by similar ultrasound irradiation to pretreated NB is only about 50% (0.24 nl/0.51 nl). This may be important evidence that <200NB is not easily disintegrated by 1 MHz ultrasound. Most of the gas involved in sonoporation is maintained in 200NB ≤. According to these results, centrifugal removal of 200NB ≤ reduces the total amount of gas in the liquid and might have affected subsequent reduction in sonoporation efficiency. This result suggests that both the size and the total gas volume of NBs are important parameters in sonoporation.

Indeed, an alternative possibility may just be that the number or concentration of NBs present surrounding the target cell affected the sonoporation efficiency. The complexity of bubble behavior during ultrasonic irradiation, especially in the case of NBs, must be taken into consideration before coming to any conclusion.

Our study demonstrated the importance NB size distribution on sonoporation for intracellular gene delivery. In order to control or maximize the efficiency of NBs for gene therapy, it is extremely important to adjust the bubble size, concentration and the total amount of gas that may determine the beneficial outcome for this therapy. Future studies need to clarify the effects of the many external stimuli factors related to this modality.

## Data Availability

The raw data supporting the conclusion of this article will be made available by the authors, without undue reservation.
