# Supplementary material for: Influence of Nanobubble Size Distribution on Ultrasound-Mediated Plasmid DNA and Messenger RNA Gene Delivery
Source: Front Pharmacol. 2022 Jun 1;13:855495. doi: 10.3389/fphar.2022.855495 (PMC9198282; doi:10.3389/fphar.2022.855495)
Supplement: Supplementary file 1 [file DataSheet1.PDF]

Supplementary Material

Supplementary Table 1. Alterations in nanobubble particle size, concentration, and total gas volume with centrifugation.

	Diameter (nm)	Concentration (/mL)	Distribution (%)		Total gas volume (nL/mL)
			<200NB[a]	200NB≤[b]	
0 g	254.7 ± 3.8	8.6×10^8	32.1	67.9	5.11
100 g	201.9 ± 0.4	4.8×10^8	63.4	36.6	1.28
5000 g	187.3 ± 4.8	2.6×10^8	69.7	30.3	0.53

[a] nanobubbles less than 200 nm

[b] nanobubbles of 200 nm or more

Supplementary Table 2. Alterations in nanobubble particle size, concentration, and total gas volume with various sonication intensities.

	Diameter (nm)	Concentration (/mL)	Distribution (%)		Total gas volume (nL/mL)
			<200NB[a]	200NB≤[b]	
0 W/cm^2	254.7 ± 3.8	8.6×10^8	30.2	69.8	6.53
2.5 W/cm^2	203.8 ± 11.3	4.8×10^8	70.3	29.7	0.71
5.0 W/cm^2	191.4 ± 14.3	2.6×10^8	74.8	25.2	0.46

[a] nanobubbles less than 200 nm

[b] nanobubbles of 200 nm or more

Supplementary Table 3. Alterations in nanobubble particle size, concentration, and total gas volume with various sonication duration.

	Diameter (nm)	Concentration (/mL)	Distribution (%)		Total gas volume (nL/mL)
			<200NB[a]	200NB≤[b]	
0 sec	254.7 ± 3.8	1.0×10^{9}	30.4	69.6	5.94
5 sec	-	2.5×10^{8}	64.7	35.3	-
10 sec	186.0 ± 9.8	1.8×10^{8}	73.5	26.5	0.36
20 sec	-	1.6×10^{8}	77.6	22.4	-
30 sec	178.9 ± 5.4	1.6×10^{8}	78.6	21.4	0.28
60 sec	-	1.2×10^{8}	78.1	21.9	-

[a] nanobubbles less than 200 nm

[b] nanobubbles of 200 nm or more

Supplementary Table 4. Alterations in nanobubble particle size, concentration, and total gas volume with combination of centrifugation and sonication..

	Diameter (nm)	Concentration (/mL)	Distribution (%)		Total gas volume (nL/mL)
			<200NB[a]	200NB≤[b]	
Cfg[c] (-), US[d] (-)	254.7 ± 3.8	9.4×10^{8}	29.4	70.6	5.58
Cfg (+), US (-)	217.1±8.0	1.5×10^{8}	64.7	35.3	0.51
Cfg (+), US (+)	163.9 ± 4.6	1.9×10^{8}	74.6	25.4	0.24

[a] nanobubbles less than 200 nm

[b] nanobubbles of 200 nm or more

[c] centrifugation

[d] ultrasound
